# Proposal of an Economy of Things Architecture and an Approach Comparing Cryptocurrencies †

**DOI:** 10.3390/s21093239

**Published:** 2021-05-07

**Authors:** Bruno Machado Agostinho, Mario Antônio Ribeiro Dantas, Alex Sandro Roschildt Pinto

**Affiliations:** 1 Computer Science Graduate Program, Federal University of Santa Catarina, Florianopolis 88040-900, Brazil; a.r.pinto@ufsc.br; 2 Computer Science Graduate Program, Federal University of Juiz de Fora, Juiz de Fora 36036-900, Brazil; mario.dantas@ice.ufjf.br

**Keywords:** blockchain, cryptocurrency, economy of things, Ethereum, Iota, Ripple

## Abstract

In the present computational scenario, one can perceive the emergence of cryptocurrencies and the increased utilization of IoT devices, which are pushing to new challenges, opportunities, and behavior changes. It is still not known how these technologies will impact the current business and economic models. In this regard, this study proposes an economy of things architecture and an approach comparing several cryptocurrencies. Therefore, the proposed architecture aims to use these new opportunities to enable device-to-device (D2D) interaction based on this novel paradigm, called the Economy of Things (EoT). An experimental environment was conducted to compare characteristics of the cryptocurrencies Ripple, Iota, and Ethereum. The initial results show several interesting differences related to transaction costs, errors, speeds, and threads.

## 1. Introduction

The significant increase in the number of devices in the Internet of Things (IoT) context and the emergence of cryptocurrencies lead to some interesting challenges, opportunities, and behavior changes. The authors in [1] envisioned that the IoT would cause an economic impact in upcoming years, potentially changing some business models as we know nowadays. However, there are still some issues that need to be solved. IoT platforms are not usually made to integrate with others.

The IoT demands soft and scalable solutions, guaranteeing security and privacy. The concept of blockchain has a great potential for integration due to its distributed, secure, and private nature. According to [2], blockchain can be defined as a distributed ledger in a peer-to-peer architecture. All connected nodes have a copy of the data without the need for a centralized database. Being developed in a distributed architecture, blockchain can be considered a purely peer-to-peer [3] system.

Blockchain opened the door to the development of an open, scalable digital economy, one that does not need third-party supervision to carry out transactions. Since its inception with Bitcoin [4], other cryptocurrencies with similar technology have emerged, in addition to other approaches. At the same time, the use of blockchain and cryptocurrencies in applications and systems is growing steadily.

It was within the context presented so far that the Economy of Things (EoT) paradigm emerged. It can be defined as the monetization of things or the possibility of exchange between digital assets. The integration between blockchains and the IoT can bring numerous advantages in this scenario. The recent launch of Industry Marketplace by IOTA Foundation is an example of an application within the context of the EoT.

Most of the proposals found in the literature [5,6,7] revolve around how to offer and consume services or data and are focused on the platform users. In contrast, the present study proposes a differentiated architecture that enables the same integration and the possibility of removing or reducing human interference as much as possible. The approach was conceived for device-to-device interaction, allowing the comparison between multiple cryptocurrencies with the potential to impact the current business models. Therefore, three cryptocurrencies were chosen to allow the experiments. Iota [8] was released to be the main IoT protocol, Ripple [9] aimed to change the way in which we send assets around the world, and Ethereum introduced Smart Contracts through blockchains.

This paper is organized as follows. Section 2 brings some relevant concepts related to the proposal. Section 3 presents some related work. Section 4 and Section 5 introduce the proposed architecture and the experimental environment. The following two sections discuss the results obtained by making a comparison between Ripple, Iota, and Ethereum, addressing the analysis of our environment. Conclusions and future work are presented in Section 8.

## 2. Cryptocurrencies and the Economy of Things

### 2.1. Cryptocurrencies

The concept of cryptocurrencies started between 2008 and 2009 with the publication and development of Bitcoin [4]. The proposal was to store data in a structure called blockchain, within a peer-to-peer system and with a consensus algorithm for asset exchange without intermediaries. With the increase of Bitcoin, several other cryptocurrencies were released in the last years, such as Ripple, Iota, and Ethereum, implementing the concept of smart contracts, which had already been proposed by [10].

#### 2.1.1. Iota

Iota is a cryptocurrency created in 2015 to be used in IoT devices communication. Unlike most other currencies, it does not use blockchain for data storage. Instead, it uses a network built on top of a directed acyclic graph (DAG) called Tangle [8].

The main difference between a DAG and a blockchain is how the data are stored. Blockchain transactions are stored in blocks, and each block references the previous one. In a DAG, each transaction is stored directly and, for the storage to take place, at least two existing transactions in the network need to be confirmed, making the system scalable. According to [11], the tangle succeeds the blockchain as its next step and offers the required features to device-to-device micropayment systems.

#### 2.1.2. Ripple

According to [9], Ripple is a payment system and a cryptocurrency that was released entirely independently of Bitcoin. Although Ripple used to be the second highest market cap after Bitcoin, there are no studies that analyze its provisions.

Different from most of the other cryptocurrencies, Ripple is capable of performing cross-currency transactions in a matter of seconds for a small fee. Its transaction time makes it an attractive technology to be used on economy domains, such as bank systems.

#### 2.1.3. Ethereum

Ethereum is a blockchain implementing Smart Contract functions and enabling some logic over the transaction we do not see in other blockchains like Bitcoin. It also can be defined as a transaction-based state machine [12]. Similar to other public blockchains, Ethereum has its transactions grouped into blocks and added to a chain. Its mining process is very similar to Bitcoin, where miners should validate the transactions, adding the block to the blockchain. Ethereum uses the consensus algorithm named proof-of-work, the same used by Bitcoin, where miners must solve a challenge to confirm the block. For the blocks added to the blockchain, miners are rewarded with Ether tokens. Ethereum introduced a new concept fee called GAS, a charge over each computation step avoiding overloading in the network. The GAS price is set by the user who creates the transaction. Higher prices can speed up the transaction’s processing time, and low prices have the risk of never being completed.

### 2.2. Economy of Things

The Economy of Things (EoT) is a new paradigm for the exchange of digital assets. This name was first used by IBM^©^ in a report highlighting how the Internet of Things has enhanced digital markets [13]. According to the latter authors, although some assets have physical limitations to digitization, there are already opportunities for economic growth and advancement.

In [14], the authors explore the discussion on how the use of the IoT together with blockchains can create new opportunities in the shared economy area. The website Slock.it^©^ also has some cases of EoT use. As an example, it shows an application through which it is possible to pay and charge electric vehicles without third parties. In [15], the authors proposed an architecture for the Economy of Things, presenting a comparison between the cryptocurrencies Iota and Ripple.

## 3. Related Work

The changes and impact of IoT and cryptocurrencies on the current economic models are still unknown. In order to compare and validate our proposal, in this section we present some related work.

According to [16], the growing number of data marketplaces is an inevitable consequence of the IoT revolution. These will emerge as a means of exchanging and monetizing data, as well as serving as a basis for new business models. The authors define their proposal as a framework for a data marketplace that consists of a decentralized architecture, using the Tangle network to stream encrypted data, and the smart contracts of the Ethereum network for negotiation between producers and consumers. Despite providing an architecture capable of exchanging assets, their proposal does not allow the use of multiple cryptocurrencies; it was not planned to be used autonomously and focuses on a user-to-device approach.

In the proposal presented in [17], the authors emphasize that the use of a Marketplace is a key success factor for the implementation of a device-to-device economy in a smart city. They also highlight the advancement of blockchain technologies, which can be used to store and exchange data generated in this environment. The proposal consists of demonstrating the creation of an interaction environment between citizens, and can be divided into two components: PEGASUS-DAEMON and PEGASUS-APP. The daemon is responsible for receiving data from the sensors and publishing them in the Iota Tangle network. The App is an application created as an extension of Google Chrome^©^, which implements the Marketplace. Through the extension, it is possible to search and buy data from sensors scattered around the city. As well as the architecture previously presented, this proposal does not allow the use of cryptocurrencies and has a focus on user-to-device applications.

Regarding a specific type of digital asset, Ref. [7] proposed a marketplace for IoT sensors data. The device owner can register their sensors along with standardized descriptions. This way, the data consumers can query and define their criteria and the available budget. The system finds the appropriate devices and provides the data on a mutual agreement. The data exchange in an IoT environment has many specific characteristics, such as the quality of the data, the time when data were generated, among others. The solution proposed by the authors seems not to fit device-to-device IoT systems once it is not safe to configure devices to buy and sell data in an autonomous way. Despite being a proposal of credibility criteria that uses delivery rate measurements, it does not include the quality of the generated data. One can infer that D2D is a natural evolution of the IoT ecosystem.

Aiming at Smart Communities systems, Ref. [6] proposed the I3 Marketplace. They used an approach that makes it possible to publish and subscribe to data from IoT devices. The agreement occurs on an end-to-end way, between the device owner and the subscriber application developer. The proposal seems to work but it does not enable D2D systems, since the focus of the proposal is on the agreement between the parties.

According to [18], it has already been predicted that IoT devices will have the ability to monetize the data they generate and/or collect. This monetization has several challenges considering automation and scalability, in addition to normally depending on centralized systems of intermediaries. The authors then propose the use of Ethereum smart contracts to monetize data automatically and systematically. Their proposal has a user-to-device approach, and similar to the previous ones, it does not allow payment using multiple cryptocurrencies.

In the work of [19], the authors start listing the current challenges in the manufacturing sector. Especially when comparing products produced in countries with lower average wages with countries with better wages. This problem brings research motivation on autonomous machine-to-machine systems, which may replace human participants in the production chain. The proposal presented focuses on communication and payment between devices. Since the traditional means of payment were considered inadequate to this demand, the authors proposed using the Iota cryptocurrency to enable M2M payments. The idea is to transform a previous work into an M2M version. The goal is that it is possible to produce a painting using acrylic paint from a given image. After prototype development and research, the authors concluded that Iota is not ready to enable an M2M or IoT economy. The main reason, according to them, is the lack of smart contracts, which would make possible the conditional implementation of rules. Furthermore, there is a concern regarding using a network coordinator, removing the benefits of decentralized decisions.

In [20], the authors started asking about who might be interested in GPS data of people using the city tram. The mobile devices used by users and sensors in buildings can generate countless data of the most varied types that can be useful in applications. According to the authors, although the opening of this data in a public way is not ethical, users, generators, and carriers should have the option of making their data available on demand through compensation that they think is fair. The proposal presented consists of an architecture called Hermes, which enables users to sell data anonymously. They can configure the price they deem necessary and the possibility to interrupt the supply whenever they want. The Hermes system works by registering the data that a user has to offer in a Marketplace. Buyers can search and request the interested data. The payment method and the data transmission take place through the Tangle network, the Iota cryptocurrency system. The Marketplace plays an essential role in the proposed architecture.

In the [21] proposal, the authors talked about the value of IoT-generated data not only for device owners, but also the potential resale value for buyers. However, the nature of this data does not fit into traditional marketplace models of static data since much of the data generated loses its usefulness over time. The lack of trust between participants in a marketplace is a challenge, and the new generation of blockchains with support for smart contracts appears as a natural choice to solve the problem. The authors propose using smart contracts from the Ethereum cryptocurrency to enable the interaction between data providers and consumers. Although they use smart contracts to register producers, consumers, and storage, the sending of data takes place directly, not using Ethereum’s blockchain resources.

In the work of [22], a multilayer marketplace was proposed for the sale and sharing of data generated by IoT sensors through blockchains. The goal is to enable manufacturers to send data to different consumers. This process would facilitate the acquisition of data, which was often not obtainable, by companies or products that wish to carry out analysis and knowledge discovery to improve their products. It would generate a kind of ecosystem where manufacturers can start monetizing their data, reducing the amount charged for their products. Artificial intelligence vendors can sell improved analytics to companies, which in turn can improve their products. Finally, end users can get products at reduced prices in exchange for providing their data. They developed a proof of concept architecture to validate their proposal. After researching possible cryptocurrencies, the authors discarded the use of Iota, since the use of network coordinators breaks the concept of decentralization. Furthermore, discarded EOS currency as the implementation would double the number of requisitions required. So, they developed using the Ethereum blockchain. The data exchange occurred through smart contracts, and the tests were considered satisfactory by the authors.

In addition to the works mentioned above, there are also proposals with similar approaches that were used for comparison with our proposal, such as the works [23,24].

## 4. The Proposed Architecture

**Figure 1:**
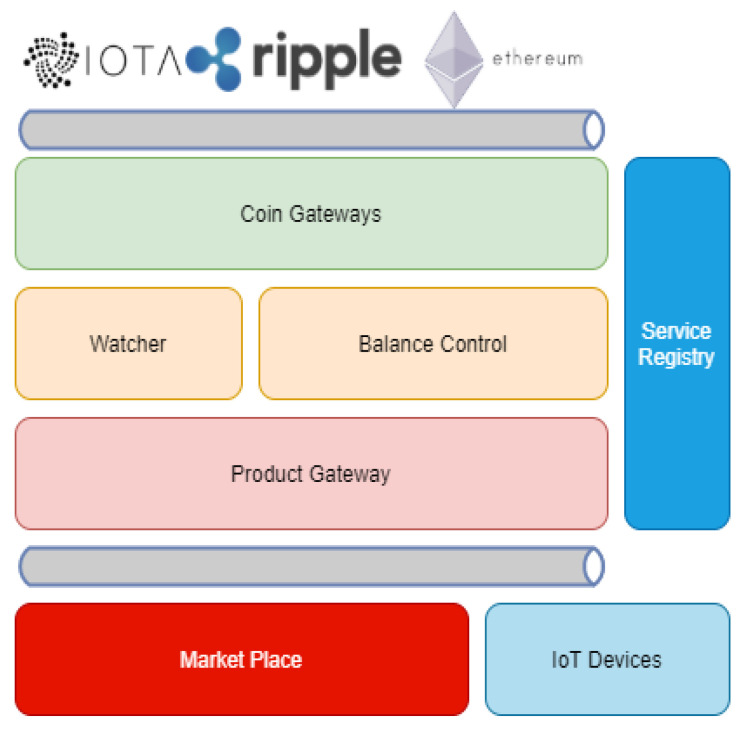
Proposed architecture.

In order to enable device-to-device interaction in an EoT context, we propose an architecture that aims to remove or reduce human interference as much as possible. Figure 1 shows the proposed architecture and its components.

We designed each module as an independent component, resulting in an approach using microservices to reach the wanted flexibility. Each component will be explained in detail for a better understanding of the proposal.

Service Registry: It is responsible for registering the microservices. There are three different registration options: Service, Device, and Crypto. The service registry module returns the correct keys for each service based on the communication needed.

IoT Devices: The device represents a producer. It means any entity that wants to sell any asset. Its role is to inform what it is producing, manage its stock, and it can be a client from the marketplace module, searching for products.

Product Gateway: It is responsible for registering the products offered in the marketplace. This module handles the interactions with the IoT devices. Requests for books, orders, and confirmations will always be performed by this module. In our proposal, an instance of the proposed architecture can have only one product gateway, but several IoT devices representing different products.

Coin Gateways: These modules are responsible for the integrations with cryptocurrencies. As shown in Figure 1, we used three modules for communication with the Iota, Ripple, and Ethereum networks.

Balance Control: Once we can use multiple cryptocurrencies, the balance control module is responsible for abstracting the communication with the coin gateways. This module makes every request to use funds.

Transaction Watcher: This is an asynchronous module with only one task: confirm cryptocurrency transactions. When a transaction finishes, the Watcher notifies the Product Gateway to confirm the order.

Marketplace: It is a module where several instances of the proposed architecture can register their devices, enabling a Provider/Consumer IoT ecosystem.

## 5. Experimental Environment

**Figure 2:**
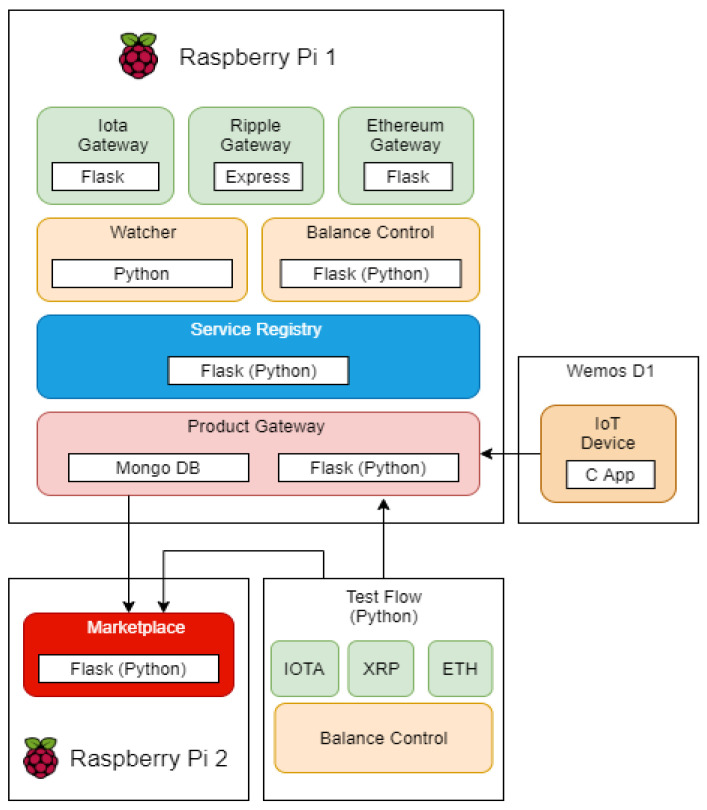
Experimental environment.

The experimental environment was conceived to test and compare the behavior of the three cryptocurrencies used in this proposal. Figure 2 shows how we used the proposed environment in the tests.

The marketplace was placed on a Raspberry Pi 2. Its prototype was developed using the framework Flask for Python 3.6. As we just used it for the tests, it has only the endpoints *register* and *search*.

The experimental environment considers a Raspberry Pi 2 together with a Wemos D1 board to represent an instance of the proposed architecture. Almost all modules were developed using the Flask framework. The exceptions were the Coin Gateway for Ripple, developed using Express.js, and the device application, developed in C. A MongoDB instance was also used, placed on the Product Gateway, to register all orders.

All Coin Gateways started the tests using the available test networks instead of configuring and using a private node. For Iota, we first need to change the tests to the main network of the tangle and then change to a paid permanode, in an attempt to avoid timeout and exceeded requests. We used the PyOTA library for Iota connections, ripple-lib for Ripple, and Web3-py for Ethereum.

Although we use all the components in the same device in the tests, the proposed architecture can be implemented in several ways. Because it is a microservice architecture, each module can be deployed in any way that the user sees fit. The limiting factors here would be the libraries available for connection to the blockchains. Since not all blockchains have libraries with support for all languages, the implementation of each module should take this into account.

### Testing Methodology

Since part of the proposed architecture uses integration with the chosen cryptocurrencies, the planning of the tests had to consider that the most critical part of the process would take place at the time of the blockchain transactions. As we do not have access to make changes to the blockchains, the tests were designed to demonstrate the viability of each cryptocurrency in an environment where there is a need to make several transactions in a short period. After consulting the literature, two types of tests were chosen. The first one is the Load Test, which aims to detect functional problems, such as deadlocks and racing, and quality criteria, such as reliability, robustness, and stability. The second, the Performance Test, which is usually used to return metrics such as response time, throughput, and resource utilization [25]. In the proposed architecture, the performance test will be used to measure the confirmation time of the transactions.

From this paragraph and on, we will refer as a performance test to all tests related to the load and performance tests mentioned in the previous paragraph. According to [26], a performance test has the following key performance targets: Availability or uptime, concurrency, scalability and throughput, and response time. As secondary targets, the authors still list the server and network utilization. For our tests, the main targets are concurrency tests and response time. Although we are aware that the network is an essential factor in our architecture, our objective is to carry out the tests to verify the integration behavior with the cryptocurrency systems.

Furthermore, according to [26], performance testing tools usually have the following components: Scripting module, Test management module, Load injectors, and Analysis module. Since our tests will have to be adapted to test an integration with third-party systems, we decided that although the tests would use the scripting part, performance tools would not be used.

We designed the test planning to simulate a situation where the architecture needed to make several micropayments through multiple devices. For this, we designed a test flow to be performed with different concurrency configurations, starting with just one thread simulating a device and ending with one hundred. Besides the number of threads, the internal flow of each device also had many repetitions, simulating several payments being made in sequence by each one of them.

**Figure 3:**
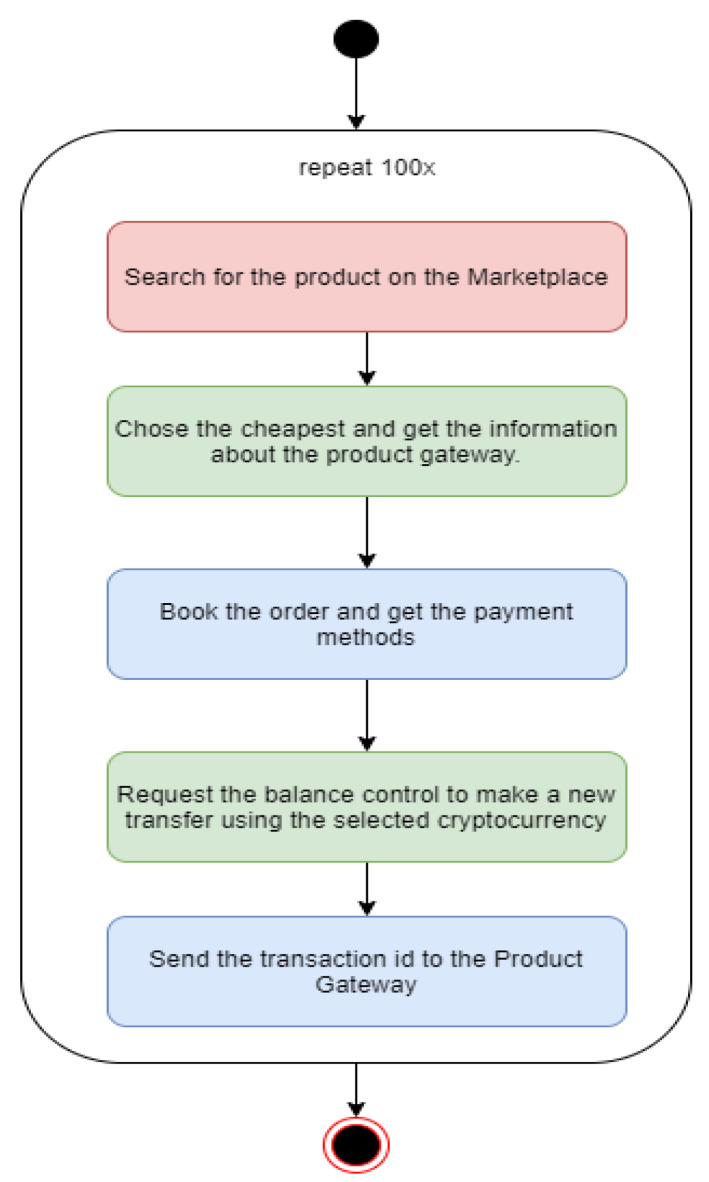
Test flow.

We used a python script to control the flow and perform the tests. The Balance Control modules and the three Coin Gateways were placed in a device in order to simulate potential buyers. The test started by using one thread executing 100 times the flow, as shown in Figure 3. We incremented the number of threads, performing the tests with 5, 10, 25, 50, and 100. The steps marked in green represent the execution on the consumer side, the ones in blue represent the producers, and the red one represents the marketplace.

All executions first used only one currency and repeated to the others. More than fifty thousand transactions were created in the process.

## 6. Results and Discussion

For a better understanding, this section unfolds into four subsections, first presenting the individual results obtained with each cryptocurrency and ultimately comparing both.

### 6.1. Iota

**Figure 4:**
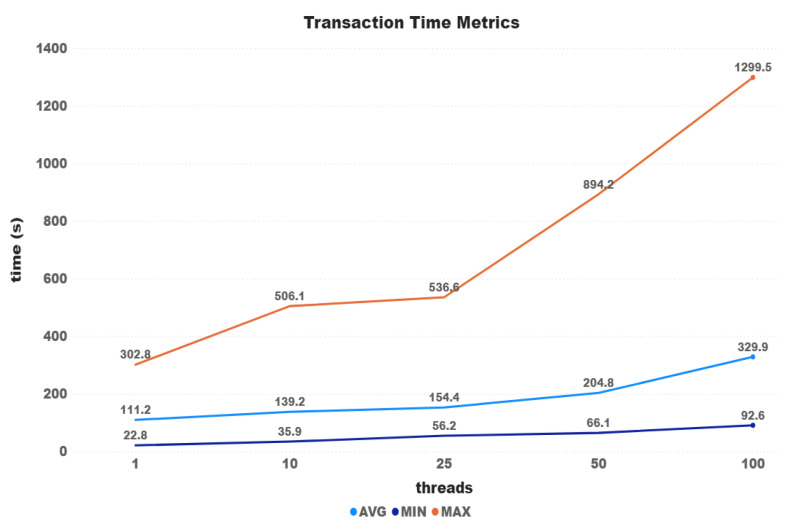
Iota time metrics.

Figure 4 shows transaction time metrics according to the number of threads used on the test. Although it is possible to see an increase between each test, time growth does not follow the same proportion of the threads. The differences between the first and last tests were 296% for the average time, 418% for the minimum time, and 430% for the maximum time. The difference between the fastest transaction (1 thread) and the slowest one (100 threads) was almost 20 min (5900%).

**Figure 5:**
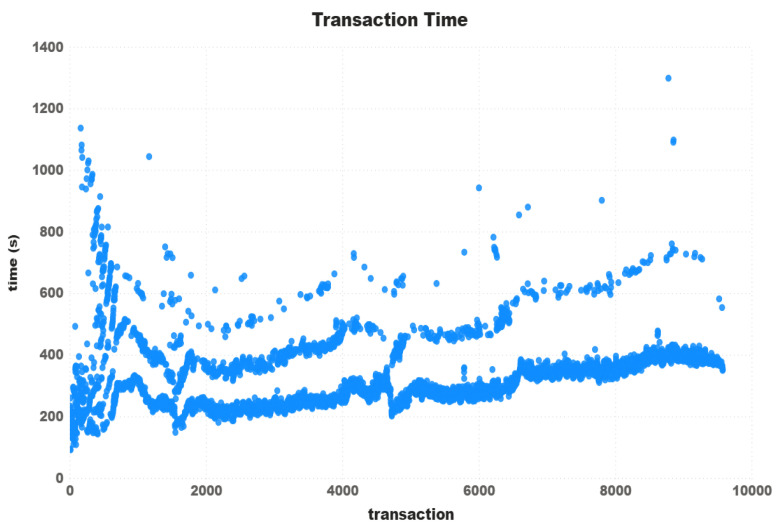
Iota transaction time—100 Threads.

With an in-depth analysis of the transaction time, one can see in Figure 5 three well-formed paths over the 9570 valid transactions. The first and most dense path started with the values slightly lower than the average of 329 s and finished a bit higher. The second one started higher than the average, had some values lower but finished with more than double the value. The third and not so dense path started higher than the previous two, but lost its density over the transactions. After 5000 transactions, it is possible to say we have only two paths.

With the increase of threads, the Iota network presented some instability. Table 1 displays the number of request errors and timeouts for each test configuration. The tests started with no errors and finished with 4.3% of unconfirmed transactions, and 14.30% of the requests needed to be re-sent.

### 6.2. Ripple

**Figure 6:**
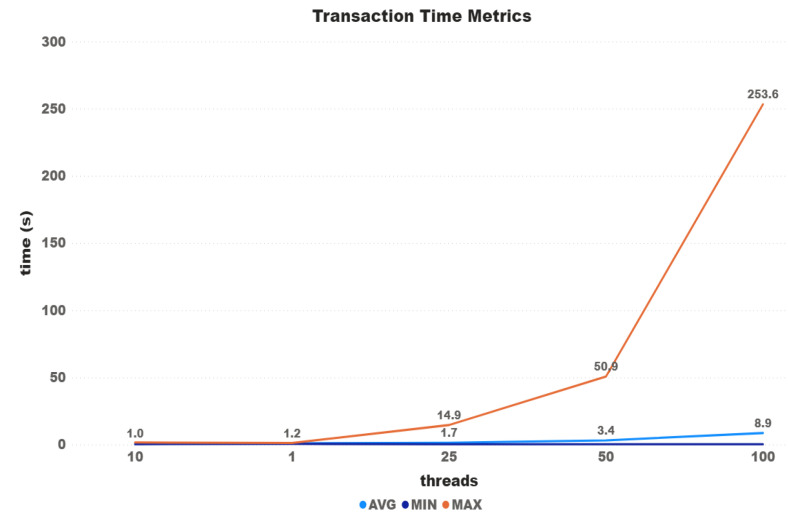
Ripple time metrics.

As can be seen, Figure 6 shows very stable values for both metrics of average and minimum times on the tests using the Ripple cryptocurrency. However, there is also a fast increase in the maximum transaction time over the 10,000 transactions. The differences found between the first and last tests were 0.75% for the minimum time, 729% for the average time, and 17,300% for the maximum time. The average metric value deserves attention since, looking at the chart at first glance, it seems much more stable than Iota. However, when using the values, the difference is more than double.

**Figure 7:**
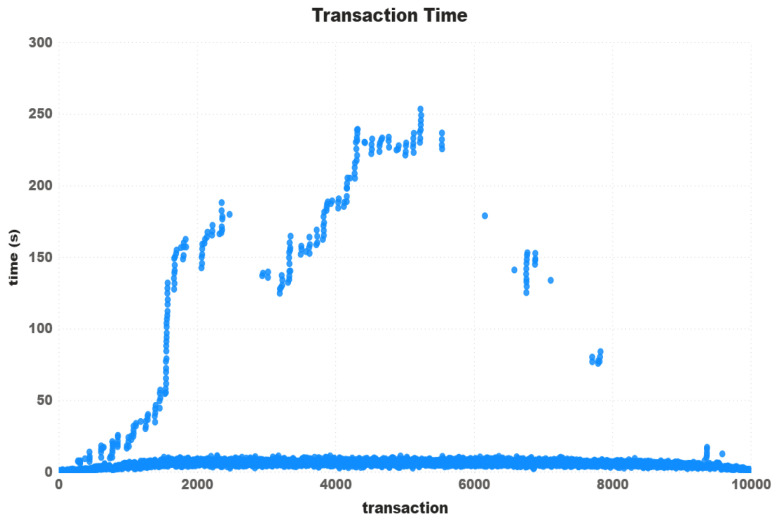
Ripple transaction time—100 Threads.

The values found on the Ripple transactions time (Figure 7) show a very different result than those found with IOTA. We can see a very dense path under the line of 12 s, almost 3 s higher than the average. There exists another path, but after 5000 transactions, it starts to turn into small clusters instead of a path. After comparing both scatter plots, it is reasonable to reckon the Ripple as the most stable coin when we use the average transaction time to compare.

Table 2 (Conversion made using the site www.worldcoinindex.com, accessed on 6 May 2021) shows the fees applied to each test configuration. Although the Ripple network charges a variable fee, one can see that such a small value does not make its use unfeasible. For the tests, we obtained an average fee of 0.000012 XRP (USD 0.000002952). The total value spent on the tests was USD 0.05490 for 18,600 transactions.

### 6.3. Ethereum

The Ethereum cryptocurrency has a distinguishing feature compared to the previously tested cryptocurrencies, which is the possibility of managing the price offered by the executed commands (GAS) in the transactions. Because it is managed by the user, its variation can influence the final price paid and the waiting time that a transaction remains in the network before being sent. However, the libraries used have the ability to inform the average network price at the time of the transaction.

**Figure 8:**
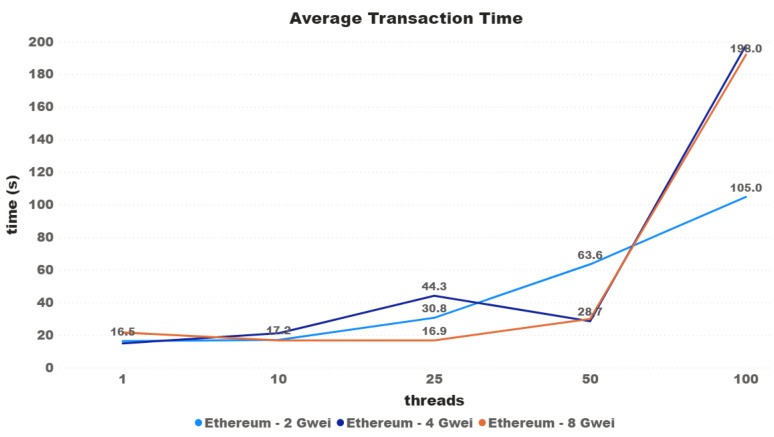
Average transaction time.

At the time of testing, the average price paid for GAS was 4 Gwei, equivalent to 9 billion Wei—the smallest possible unit in the chain. For the Ethereum network testing, the assumption first raised was that transactions would take longer the lower the price, and less time the higher the price. Initially, it was decided that the tests would be carried out with the current network average value (4), with double and with half of that value. Figure 8 shows the results of the first test performed. As can be seen, it cannot be concluded that this difference in the amount paid actually influences the average time. For the 100-thread test, the lowest-priced transactions were completed in almost half the time of the other two. It was then decided to increase this difference, now using the prices of 0.4, 4, and 40 Gwei. For the tests with 0.4 and 100 threads, we had almost 10% of transactions that never finished or that were invalid on the Ethereum network test.

**Figure 9:**
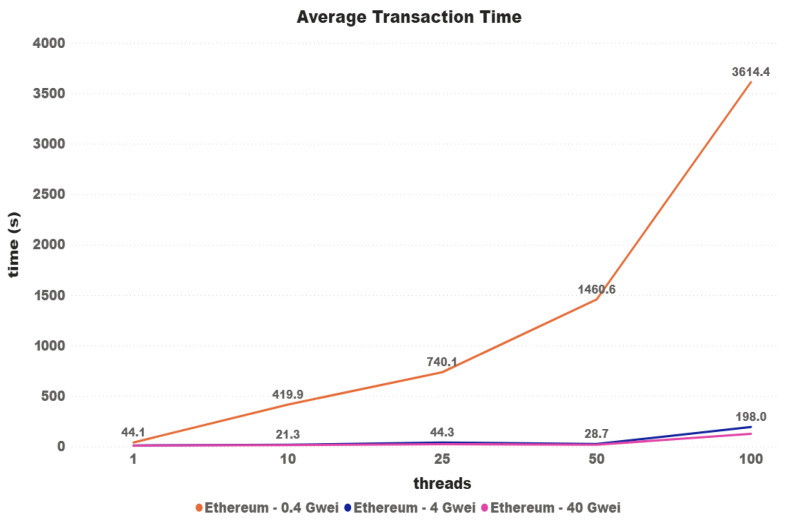
Average transaction time.

In Figure 9, it is possible to notice that, for the price of 0.4 Gwei, there was an interference in the time of the transactions, reaching a difference of more than 1825% in relation to the tests with other prices. In the comparison between the prices of 4 and 40 Gwei, the initial difference started at approximately 15% and ended at 51.56%, leading to the belief that such a big price difference can really guarantee that transactions are finalized faster.

**Figure 10:**
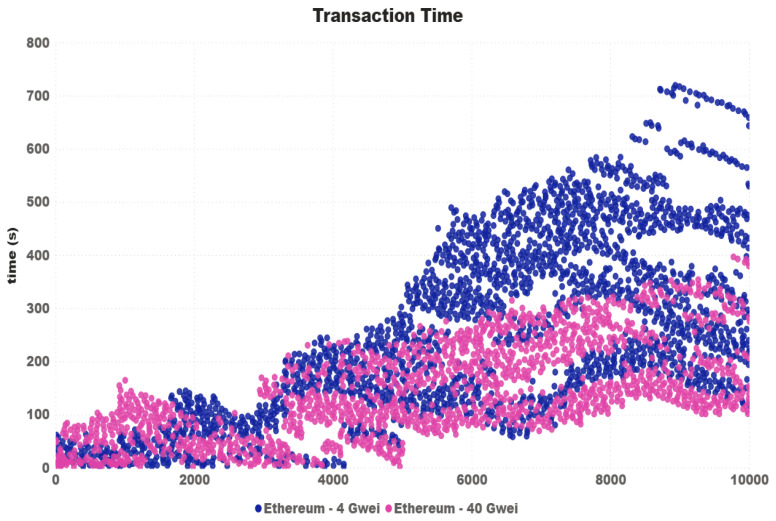
Transaction time.

Looking at the transactions more closely, Figure 10 shows the waiting time of all 10,000 transactions carried out in the 100-thread test for the prices of 4 and 40. Although it is clear that, for the configuration of 40 Gwei, transactions on average are really faster, at different times the waiting times for 4 Gwei were shorter.

Another hypothesis raised is that price setting could then have a greater impact on the maximum waiting time for transactions. The minimum time has been discarded since this has a direct influence on the minimum time for a new block to enter the network.

**Figure 11:**
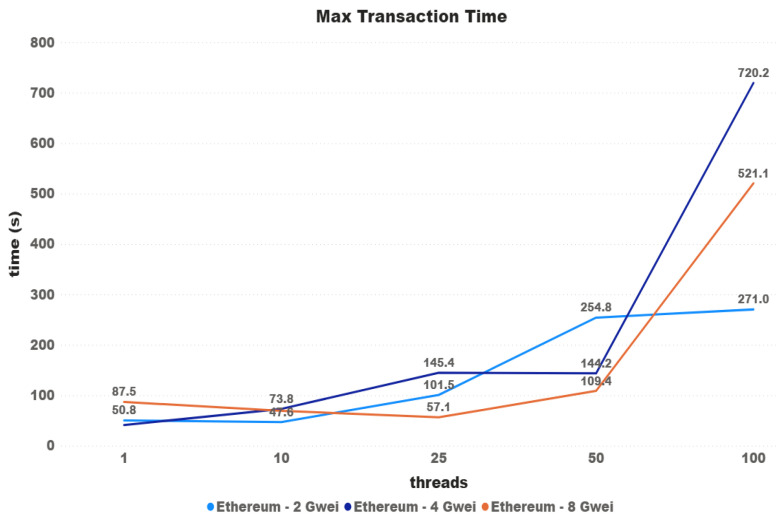
Max transaction time.

**Figure 12:**
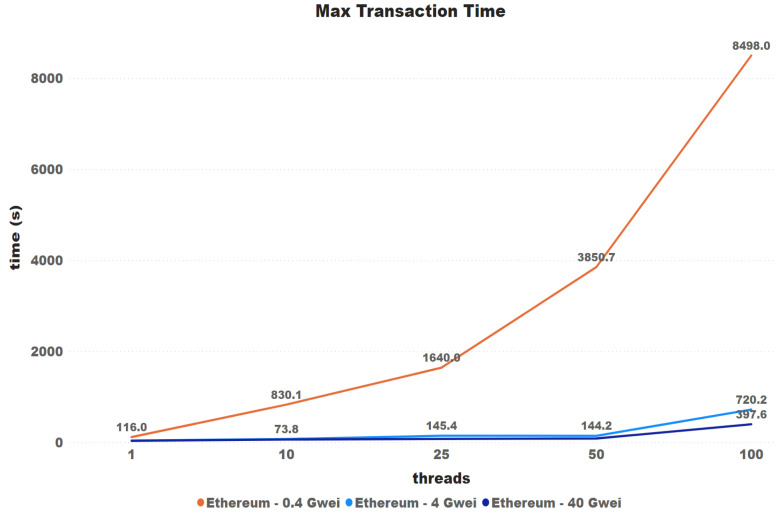
Max transaction time.

Figure 11 and Figure 12 again show that there is a misunderstanding for large differences in price, i.e., there is a certain influence, which is not proportional to the difference in values and could not be confirmed for minor differences. The difference between the 4 and 40 Gwei price settings started at 23.70% and ended at 81.14%.

Table 3 shows the approximate costs achieved by testing 100 threads with each of the price configurations. Starting with approximately 0.027 USD per transaction, the tests ended with a value of 2.7 USD.

### 6.4. Iota vs. Ripple vs. Ethereum

**Figure 13:**
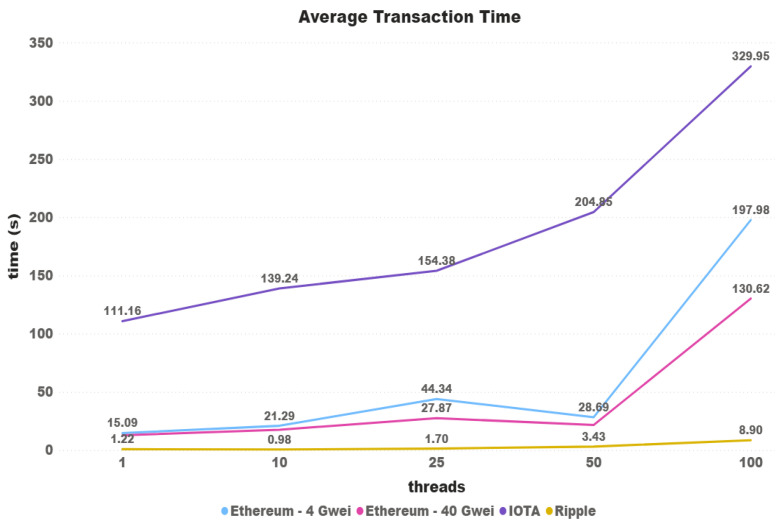
Iota x Ripple: Average transaction time.

Figure 13 shows a transaction time comparison between the three cryptocurrencies regarding the increasing number of threads. As can be seen, Ripple had the lowest values over all tests, and Iota had the highiest ones. Starting with 9111% slower than Ripple and 736% than Ethereum (4 Gwei), the difference between the average time decreases (except with 10 threads) until the difference of 3707% between Iota and Ripple, and 197.98% between Iota and Ethereum (4 Gwei) with 100 threads.

**Figure 14:**
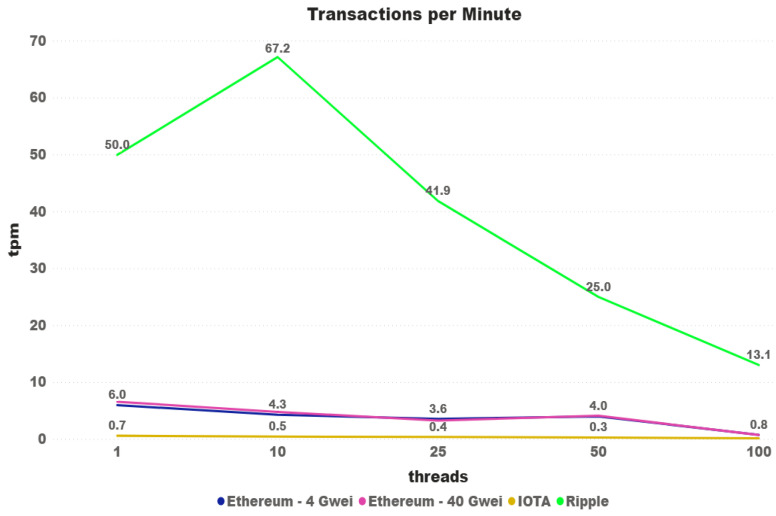
Iota x Ripple: Transactions/minute.

Looking at the same information from a different perspective, Figure 14 shows the transactions confirmed per minute of both cryptocurrencies. In the last graph, it is clear to see that the difference decreases over the tests. A surprising increase happened on Ripple tests with 10 threads, obtaining more than one transaction confirmed per second.

It is worth emphasizing that we used an over-dimensioned number of threads in an attempt to simulate a real environment. It is unlikely to see 100 different devices doing 100 sequential orders for the same device. Maybe if we kept the number of threads increasing, we could have obtained similar times for both currencies. There is a concern about how much time each transaction could take in this scenario.

### 6.5. Discussion

Before conducting a more in-depth comparison of the results obtained, it is worth remembering that the three cryptocurrencies chosen for the tests have very different characteristics, especially concerning the protocol used to confirm transactions on the blockchain. Although they were chosen because they fit what we consider necessary within an environment implementing the Economy of Things paradigm, they have very different characteristics.

As a semi-private blockchain, Ripple was expected to have the best confirmation times. Since they have private nodes to confirm transactions, there is no need to wait for the spent time with consensus protocols such as Proof-of-work (PoW). PoW is used on blockchains where the lack of trust between users of the blockchain is resolved through challenges of high mathematical complexity leading to competition between users for the right to insert the block into the blockchain. Despite the lack of studies on performance in confirming transactions, they are supposed to occur instantly since the objective of this cryptocurrency is the immediate transfer of assets between parts around the world.

The cryptocurrency Ethereum is known to be one of the first to implement a layer above the blockchain that functions as a virtual machine, where it is possible to create smart contracts. In addition to smart contracts, Ethereum has conventional token transactions and uses PoW as a consensus protocol. Although Ethereum blocks are created periodically, this cryptocurrency has a feature that directly impacts the transaction confirmation time, the concept called GAS. Each Ethereum transaction has a set of steps, and each step is called GAS. To insert a transaction on the network, the user should define the amount paid for each step. This definition is a critical step, as high amounts paid can make the transaction be confirmed faster and waste tokens, while lower amount transactions can never be confirmed. As it is possible to see in [27], between 2017 and 2019, the average time of the blocks was near of 15 s. Figure 8 shows that the average time for requests with only one thread, using the network’s average GAS price, was within the expected, making the transaction be placed in the first two blocks. Even though in some cases the average for transactions with a lower GAS value was lower, in Figure 9 it is possible to see the behavior when placing a GAS value that is too low.

Unlike the other two mentioned above, Iota does not use a blockchain itself but a DAG. It also makes the message confirmation protocol very different. Instead of using blocks containing transactions, each transaction is inserted directly on the Tangle network. For each transaction placed on the network, the user must confirm at least two others. It is supposed to make the network faster since the more transactions are placed, the faster they are confirmed. Until now, the Tangle network still has a network coordinator to assist in confirming messages, causing Iota to be no longer chosen for use in some cases, as it is possible to see in some works listed in Section 3. In the work of [28], the authors collected transaction data from the Tangle network to recreate it and perform some empirical tests. The results showed that only 5% to 12% of the transactions took less than 1 min to confirm, and 65 % to 85 % took 1 to 10 min. It is possible to see in Figure 4 that the results found for all test configurations reached the time range of 1 to 10 min. For the tests with 100 threads, it is possible to see in Figure 5 that although the majority of requests were below 10 min, the tests took more time in several cases, especially in the first transactions. Furthermore, we also discarding those transactions that were not confirmed within the proposed time.

Although the characteristics of each cryptocurrency presented above can change, or even improve over time, the data presented here can be used as a reference for future decision making in projects where the options for transaction confirmation protocol are still being evaluated or even for choosing similar cryptocurrencies. The choice of the protocol will impact more on the confirmation time than on the proposed architecture itself. Despite the fact that the architecture can use devices powered by a battery, it is possible to configure them to sleep and check the transactions periodically, avoiding being susceptible to the transaction time.

## 7. Environment Analysis

This section will address the challenges and drawbacks that we had in our experimental environment to finish the tests.

### 7.1. Ripple

The first problems that we had with the Ripple network were slow transactions and connection errors. In the tests with 1 thread, the average time was 9 s. This value is almost seven times higher than that of the final tests. To solve the problem, we started to re-utilize the connection. We changed from opening the connection at every request to a unique connection for all Express endpoints. After the changes, the average time decreased to 4 s.

We also had a problem with duplicated and invalid transactions. It was solved by using a random value for the transaction, as we started using different addresses in the Ripple network for each thread.

The most significant issue on Ripple was the problem of confirming the transactions after some time and some ledger version errors. To solve it, we started using the status returned on the transaction as the confirmation status, thus creating a second and sequential confirmation step on the IoT device. With this last change, we reached out to the presented times.

### 7.2. Iota

As experienced with Ripple, the first problem found in the Iota network was the slow requests in the network. We realized that it might have been caused by the use of a fixed address. First, we changed towards generating a new address every transaction, and then we used a thread to change the addresses every 10 s.

After slow results and timeouts, we changed the tests from the developer to the Main Iota network. However, for 50 and 100 threads, we started to exceed the requests allowed. Therefore, we changed to a paid Permanode. It is worth stressing that we were able to set up our own node. We will not count the use of a paid node as a cost from the Iota network.

While performing the tests, we noticed that some transactions were not confirmed. First, we tried to reduce the Proof-of-work complexity on the transaction. This solution started to create invalid transactions, so to solve the problem, we started counting any transaction that took more than 1 h to confirm as a timeout.

### 7.3. Ethereum

The first difficulty encountered in the tests with Ethereum was in relation to setting up the experimental environment. The gateway for connection with the Ethereum network was developed by using the Ganache application, which creates a private Ethereum network and allows the configuration of several parameters, including the number of users and the balance of each one. The problem arose when it came to testing what was developed in a public Ethereum network. Based on the values presented above, it is clear that it is not feasible to carry out these tests on the main Ethereum network due to the cost. The test network, called Ropsten, was then chosen. It was observed that, similar to the Ethereum network, Ropsten only provides users with a balance of 0, requiring mining to obtain Ether. There are tools, such as Metamask, that can manage accounts in the Ethereum networks and have a Faucet for distributing Ether in the Ropsten network. However, the service remains down a large part of the day, and when it works, it allows the insertion of 1 or 2 Ethers before blocking the IP.

Some unsuccessful mining attempts have been made. It is not yet known if the problem was the machine used or some configuration that was missing. The mining console showed that the tasks were being carried out correctly but, even after days of mining, the balance remained intact. The only solution was to use the Metamask Faucet. It was then discovered that at certain times of the day, it was possible to get up to 10 Ethers without being blocked. After a few days of testing, it was possible to obtain 10 accounts configured with 20 Ether each.

The tests had to be carried out with multiple accounts because when entering a new transaction, each account is responsible for entering a value called *Nonce*, which consists of the number of transactions already carried out by that account. Although the libraries used provided the tools for the calculation, on the tests with several threads this value was lost, requiring the use of several accounts in a circular linked list, varying the account used in each transaction. We also had some problems to finish the test with 100 threads for the price of 0.4 Gwei. Some transactions never finished and others just did not exist in the network.

## 8. Conclusions and Future Work

In this study, we proposed an architecture targeted at enabling device-to-device interaction in Economy of Things environments. Experimental test cases were carried out to compare the characteristics of the cryptocurrencies Iota, Ripple, and Ethereum. This choice was made based on their primary goals: an IoT protocol for Iota, fast asset exchange around the world for Ripple, and the main Smart Contracts cryptocurrency for Ethereum.

The tests were conducted using the coins in an isolated way, performing 100 sequential requests per thread. We started with only 1 thread and repeated it for 10, 25, 50, and 100 threads. At the end of the tests, more than 50,000 transactions were made.

Regarding the experiments, the initial results showed that the Iota transaction time was 9111% slower than that of Ripple, and 736% slower than that of Ethereum. After increasing the number of threads up to 100, this difference decreased to 3707% and 197.98%. In addition, Iota presented some instability during the tests. The number of timeouts (confirmation time higher than 1 h) and request problems were increasing in a higher proportion than the threads. These numbers reached out to 4.3% of unconfirmed transactions, and 14.3% of requests re-sent.

Furtheremore, based on the transaction time chart in Figure 5, Figure 7 and Figure 10, one can see that Ripple had a more stable “path” on the transaction time. We concluded that, even charging some fees, the Ripple network has a higher potential to be used in IoT devices transactions. Taking into account the time rate and the network stability, we can affirm that at least for now, the Ripple network is best qualified for use on a real IoT ecosystem. Despite the fact that Iota had the highest transactions times, we consider it more viable than Ethereum, at least for now. To reduce the costs of using Ethereum, we need to start having the same instability found in Iota, with the difference that Iota is free.

As future work, additional experimental efforts will be made towards testing other cryptocurrencies. Another step forward is to thoroughly scrutinize cryptocurrency networks and try to use them as a communication approach between IoT devices.

## Figures and Tables

**Table 1:** Errors and timeouts.

	1 Threads	10 Threads	25 Threads	50 Threads	100 Threads
**Timeouts**	0	13	19	173	430
**Request Erros**	0	3	152	617	1430

**Table 2:** Transactions fees.

	100 ts	1000 ts	2500 ts	5000 ts	10,000 ts
**XRP**	0.0012	0.012	0.03	0.06	0.12
**$ (1 XRP = 0.246)**	0.000246	0.00246	0.00738	0.1476	0.02952

**Table 3:** Ethereum costs (10,000 Transactions).

	0.4 Gwei	2 Gwei	4 Gwei	8 Gwei	40 Gwei
**ETH**	0.084	0.42	0.84	1.68	8.4
**$ (1 ETH = 322)**	27.05	135.24	270.48	540.96	2704.8

## Data Availability

Not applicable.
